# The Antagonistic and Synergistic Role of Fe^3+^ Compounds in Chemo- and Electrochemotherapy in Human Colon Cancer In Vitro

**DOI:** 10.3390/ph17050651

**Published:** 2024-05-17

**Authors:** Wojciech Szlasa, Wiktoria Mazurek, Anna Szewczyk, Nina Rembiałkowska, Joanna Tunikowska, Julita Kulbacka

**Affiliations:** 1 Medical University Hospital, Borowska 213, 50-556 Wroclaw, Poland; wojciech.szlasa@outlook.com; 2 Department of Molecular and Cellular Biology, Faculty of Pharmacy, Wroclaw Medical University, 50-556 Wroclaw, Poland; a.szewczyk@umw.edu.pl (A.S.); nina.rembialkowska@umw.edu.pl (N.R.); 3 Faculty of Pharmacy, Wroclaw Medical University, 50-367 Wroclaw, Poland; wiktoria.stanco@interia.pl; 4 Department of Immunology and Bioelectrochemistry, State Research Institute Centre for Innovative Medicine, 08410 Vilnius, Lithuania; 5 Department of Surgery, Faculty of Veterinary Medicine, Wroclaw University of Environmental and Life Sciences, 50-356 Wroclaw, Poland; joanna.tunikowska@upwr.edu.pl

**Keywords:** colon cancer, electroporation, nanosecond pulses, 5-fluorouracil, iron(III)–EDTA, iron(III) citrate, frataxin

## Abstract

Colon cancer (CC) management includes surgery, radio- and chemotherapy based on treatment with 5-fluorouracil (5-FU) or its derivatives. However, its application is limited to low-grade carcinomas. Thus, much research has been conducted to introduce new techniques and drugs to the therapy. CC mostly affects older people suffering from cardiac diseases, where iron compounds are commonly used. Ferric citrate and iron (III)–EDTA complexes have proven to be effective in colon cancer in vitro. This study aimed to determine the potency and action of iron-containing compounds in colon cancer treatment by chemo- and electrochemotherapy in both nano- and microsecond protocols. The viability of the cells was assessed after standalone iron (III) citrate and iron (III)–EDTA incubation. Both compounds were also assessed with 5-FU to determine the combination index. Additionally, frataxin expression was taken as the quantitative response to the exposition of iron compounds. Each of the substances exhibited a cytotoxic effect on the LoVo cell line. Electroporation with standalone drugs revealed the potency of 5-FU and iron(III)–EDTA in CC treatment. The combination of 5-FU with iron(III)–EDTA acted synergistically, increasing the viability of the cells in the nanosecond electrochemotherapy protocol. Iron(III)–EDTA decreased the frataxin expression, thus inducing ferroptosis. Iron(III) citrate induced the progression of cancer; therefore, it should not be considered as a potential therapeutic option. The relatively low stability of iron(III) citrate leads to the delivery of citrate anions to cancer cells, which could increase the Krebs cycle rate and promote progression.

## 1. Introduction

Colon adenocarcinoma (CC) is the third leading cause of cancer, affecting mostly patients older than 50 years of age [1]. Its progression is described by the initial atypia in the normal colon epithelium [2]. The changes are associated with mutations in adenomatous polyposis coli (APC) and β-catenin genes, malfunctioningormal epithelial adhesion-mediated signaling pathways [3]. The mutations originate from the trisomy of chromosome 7, as well as the loss of the short arm of the fifth chromosome [4,5]. Further stages of carcinogenesis lead to the formation of aberrant crypts as the result of K-Ras protooncogene activation [6,7]. The next stage is the formation of an early adenomatous polyp; the morphology change is a result of SMAD4 malfunction (18q loss) [8,9]. As a result of further mutagenesis—the gain of the chromosome 20 fragment or the loss of the 17q region—the telomerase is activated [10,11,12]. Moreover, p53 loses its function, leading to the formation of late adenomatous polyps [13,14,15]. The last stage of carcinogenesis is the further dysregulation of the SMAD4 gene, resulting in the formation of invasive carcinoma [16,17]. The liver is the first organ to which CC metastases [18,19]. With the vena portae, cancer cells flow into the organ and form small pea-shaped metastatic centers [18]. When the cancer cells are present in high amounts in the blood flow, metastasis to the lungs occurs as well [20]. However, CC also causes metastases locally in the gastrointestinal organs and the peritoneum [21,22].

Ferroptosis is a non-apoptotic or necrotic type of cellular death [23]. The induction of lipid peroxidation characterizes the process as the result of Fenton’s reaction [24]. High concentrations of iron(II) ions in the cytoplasm of the cells are a factor that enhances the process [25,26]. It has been proven that ferroptosis may be used as the target point in cancer therapy [27,28]. Although the process has already been extensively characterized, it remains not fully clear [29]. One of the most unclear points is the role of frataxin in the process [30,31]. In cells, frataxin acts as an iron buffer, protecting the cancer from iron overload and supporting the cells’ energy metabolism by increasing the Fe-S clusters containing protein assemblies [31]. A decrease in frataxin expression has been proven to be a ferroptosis-enhancing factor. In vitro research has proven that the knockout of the FXN gene sensitizes the cells to ferroptosis-inducing signals [32]. The most significant problem in applying ferroptosis as an in vivo anticancer target point is the physiologically low uptake of iron ions from the gastrointestinal (GI) tract to the circulation [33]. Iron transport into the blood involves the initial reduction of Fe^3+^ ions to Fe^2+^ by the DcytB membrane-associated enzyme [34]. Further, the reduced ions are transported from the apical side of the enterocytes to the cytoplasm with the DMT1 transporter [35]. In the cytoplasm, ferritin accumulates the ions (~4000 ions per molecule) [36]. Depending on the organism’s iron demand, ferroportin (FPN) releases the Fe^2+^ ions to the extracellular matrix [37]. Further, hephaestin oxidizes iron (II) to Fe^3+^ ions. In this form, the ions bind with apo-transferrin and are delivered to distinct parts of the body [38].

Nowadays, researchers seek to enhance the cytotoxic activity of common chemotherapeutics by combining them with other anticancer compounds [39]. The other cytotoxicity-enhancing strategy is to increase the inflow of the drug into the cancer cells. For instance, with the application of ultrasound (sonoporation) or the application of an electric field (electroporation), the cell membranes become more permeable; therefore, the cells absorb greater amounts of the drug [39,40]. Superficial regions of the body, like the skin, oral cavity, rectum, and colon, are the most suitable for the application of membrane poration techniques [41,42,43]. Moreover, electroporation-based methods are efficiently used in clinical practice as electrochemotherapy (ECT), which was developed according to the European Standard Operating Procedures for Electrochemotherapy (ESOPE) [44,45,46].

According to the directives of the European Society for Medical Oncology (ESMO) from 2016, 5-fluorouracil (5-FU) combined with oxaliplatin and irinotecan is approved as the standard for metastatic CC chemotherapy [47]. Although the strategy is effective, a high level of systemic toxicity occurs [44]. Therefore, research focuses on toxicity in local tumor environments [47]. Due to the growing CC resistance and mortality, increasing research is being focused on combining standard chemotherapeutics with anticancer compounds to establish more effective therapy options [48,49]. Generally, effective compounds are those that exert a standalone antiproliferative effect among cancer cells [50]. Iron (III) citrate and iron (III)–EDTA complexes have been proven to burden the tumor in murine models of CC [51].

This study aimed to investigate the potency of electrochemotherapy with 5-FU, iron (III) citrate, and iron (III)–EDTA complexes in the induction of ferroptosis in CC cells. First, the standalone cytotoxic effect of the compounds was established. Further, the viability of the cells after incubation with a combination of 5-FU with iron (III) citrate or an iron (III)–EDTA complex was investigated. To validate the usability of drug inflow-enhancing techniques in CC treatment, LoVo adenocarcinoma cells underwent electrochemotherapy with the analyzed compounds. Additionally, the intracellular distribution of frataxin expression was taken as a quantitative measure of ferroptosis induction.

## 2. Results

### 2.1. Chemical Analysis of Composition of Iron (III) Citrate and Iron (III)–EDTA Solutions

The solutions of both analyzed compounds differed in the fraction of complexed iron and non-complexed iron forms. The iron (III) citrate solution comprised non-complexed iron and free citrate ions (Table 1). Conversely, in the iron (III)–EDTA solution, all iron was complexed with EDTA^4−^ ions.

### 2.2. MTT Assay—Drug Exposure and Interaction

**Figure 1:**
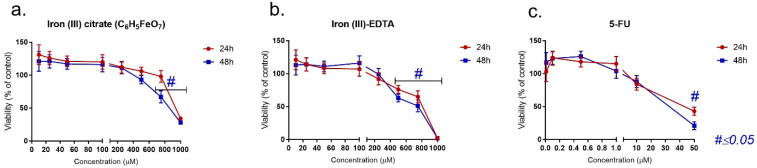
MTT assay on LoVo cells following incubation with (**a**) iron (III) citrate, (**b**) iron (III)–EDTA and (**c**) 5-FU.

The impact of three of the analyzed compounds on the LoVo cell line is presented in Figure 1a–c. In all cases, decreasing cell viability was observed with the increasing drug concentration. Fe(III)–citrate revealed a significant reduction in mitochondrial activity for the 750 and 1000 μM concentrations, and Fe(III)–EDTA reduced the viability starting from 500 μM. Although a similar pattern was observed after 5-FU treatment, the cytotoxic concentrations were about 25-fold lower than for the Fe(III) compound. A notable viability reduction was observed from the 10 μM concentration and was statistically significant in the case of 50 μM. In the case of longer exposure (48 h), only a slight decrease in cell viability was observed in each case.

**Figure 2:**
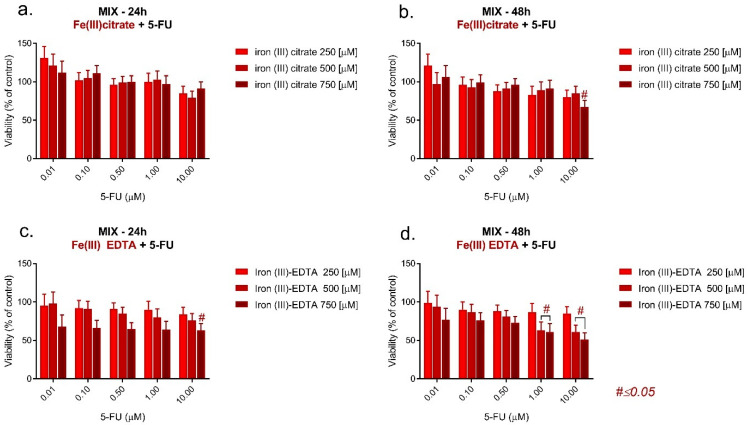
MTT assay on LoVo cells following incubation with the mixtures of iron (III) citrate and 5-FU for (**a**) 24 h and (**b**) 48 h and with iron (III)–EDTA and 5-FU for (**c**) 24 h and (**d**) 48 h.

When combining 5-FU with Fe(III) citrate or the Fe(III)–EDTA complex, the cells’ viability slightly decreased. In each case, the lowest viability (60% in 24 h incubation) was observed after combining 5-FU with 750 μM of the Fe(III)–EDTA complex. At a 24 h incubation time (Figure 2a–d), the viability was not dependent on the 5-FU concentration, and, at 48 h incubation, the increase in the 5-FU concentration led to an increase in cell viability. Although the plot pattern was not changed when lowering the Fe(III)–EDTA complex concentration to 500 μM, the mitochondrial activity increased. When further lowering the Fe(III)–EDTA complex concentration to 250 μM, the tendency was the same. However, there was no increase in cell viability observed after a 48 h incubation time. Conversely, after incubation with Fe(III) citrate, the viability of the cells was highly dependent on the 5-FU concentration. Generally, the increase in the Fe(III) citrate concentration acted protectively on the cells, increasing the viability of the cells. Irregularity in this tendency could be observed for the 1 and 10 μM 5-FU concentrations at 24 h incubation.

According to the viability measurements, the type of drug interaction was analyzed and the combination index (CI) was calculated. The results revealed that the cooperation between the dugs was antagonistic (Table 2). Both iron compounds, in the case of all used concentrations, diminished the effect of 5-fluorourcacil.

### 2.3. Membrane Electropermeabilization by Flow Cytometry

**Figure 3:**
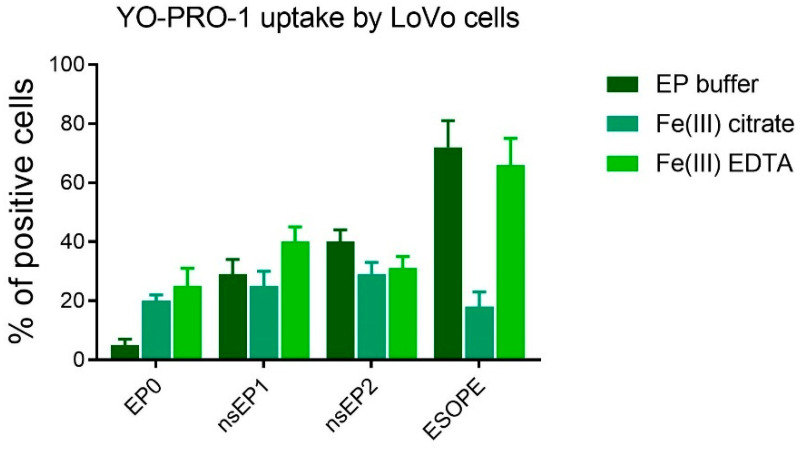
YO-PRO-1 uptake by LoVo cells treated with different agents: EP buffer, Fe(III) citrate, and Fe(III)–EDTA. Experimental conditions included treatments with nSEP1, nSEP2 and ESOPE protocols: E0—no electroporation; nsEP1—30 kV/cm, 400 pulses, 20 ns, 100 Hz; nsEP2—60 kV/cm, 400 pulses, 20 ns, 100 Hz; ESOPE—1200 V/cm, 8 pulses, 100 µs, 1 Hz.

Due to the use of the electroporation method to increase the cytotoxic efficacy of the iron compounds and 5-FU, firstly, the level of permeabilization was verified by an impermeable fluorescent Yo-Pro-1^®^ (YP1) dye. The highest cell membrane permeability was observed after the µsEP protocol (Figure 3), which is also effectively used in electrochemotherapeutic procedures [49]. The parameter nsEP-1 slightly increased the cell permeabilization level in comparison to the controls, except for 40% permeabilization with the iron (III)–EDTA complex. In the case of nsEP-2, the permeabilization level was 40% for cells in EP buffer and ca. 30% when iron compounds were added. The uptake of the others was comparable to that obtained in nsEP-1. Interestingly, for the ESOPE parameter, the permeabilization level was the highest without the use of the drugs (72%) and also increased to 66% in the presence of iron (III)–EDTA. When adding iron (III) citrate to the electroporated cells, the uptake drastically decreased to 18%, becoming comparable to the control (standalone YP1 incubation).

### 2.4. Mixed Electrochemotherapy in Colon Cancer Efficacy by Viability Assay

**Figure 4:**
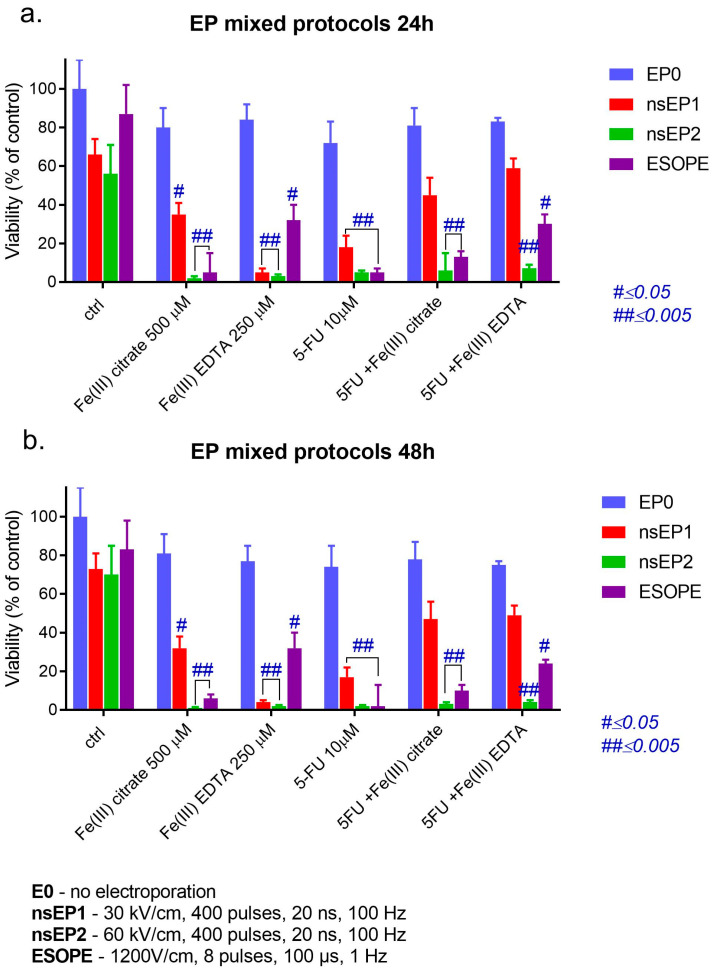
MTT assay on LoVo cells following electroporation with the mixtures of iron(III) citrate and 5-FU or with iron (III)–EDTA and 5-FU for (**a**) 24 h and (**b**) 48 h, where E0—no electroporation; nsEP1—30 kV/cm, 400 pulses, 20 ns, 100 Hz; nsEP2—60 kV/cm, 400 pulses, 20 ns, 100 Hz; ESOPE—1200 V/cm, 8 pulses, 100 µs, 1 Hz.

In the next stage, in vitro electroporation protocols were performed to determine whether iron compounds can affect 5-FU’s cytostatic effect. The most effective were the nanosecond protocols, and the most effective therapy was nsEP-2, leading to the average viability oscillating by a small percentage In almost all cases, the combinational therapies were slightly less effective than ECT with a single compound (Figure 4a,b). This observation was noted for all analyzed therapies and incubation times. The Fe(III) citrate complex alone or with 5-FU was less effective in each EP protocol and least effective in the µsEP protocol, which might indicate the slightly protective role of iron ions. The Fe(III)–EDTA complex-mediated nanosecond ECT was highly cytotoxic for colon cancer cells, and exposure to the ESOPE protocol resulted in a viability decrease for more than 60% of the cells for a single drug and ca. 40% for mixed drugs. Ranking the protocols according to their effectiveness, we can indicate the following order: nsEP-2 > µsEP > nsEP-1.

A relevant observation is that Fe(III) compounds alone do not affect colon cancer cells. Electroporation-mediated therapy significantly increases the cytotoxic effect of iron compounds, 5-FU and their combinations. According to the viability results, the drug interaction type and CI were calculated and are shown in Table 3. The calculated interaction type demonstrated that EP caused a synergistic effect for almost all mixed drug combinations. Only Fe(III)–EDTA with 5-FU exposed to nsEP1 demonstrated an antagonistic effect after both incubation times.

### 2.5. Mixed Electrochemotherapy in Colon Cancer, Fluorescent Staining of Frataxin

**Figure 5:**
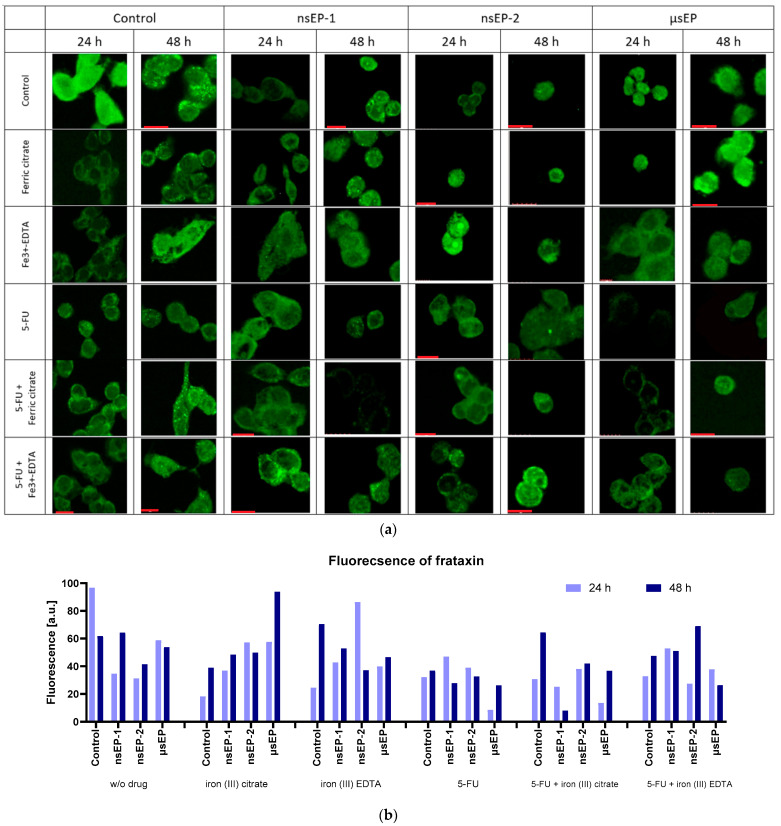
Frataxin immunostaining studies (**a**) and frataxin fluorescence analysis by ImageJ ver. 1.53 (https://ij.imjoy.io/) (**b**), where E0—no electroporation; nsEP1—30 kV/cm, 400 pulses, 20 ns, 100 Hz; nsEP2—60 kV/cm, 400 pulses, 20 ns, 100 Hz; ESOPE—1200 V/cm, 8 pulses, 100 µs, 1 Hz. Red scale bars correspond to 10 µm.

Some studies have indicated that iron complexes could reduce the fraxin levels and thus initiate ferroptosis [50], while increasing frataxin expression reduces cell proliferation and growth [51]. At present, the exact mechanisms are not well known. Thus, frataxin’s expression in colon cancer cells was visualized after exposure to electroporation-supported drug delivery. Our results showed the highest frataxin protein expression for the control cells, when no drug was added (24 h). With the increase in the incubation time to 48 h, the level of frataxin expression increased in most cases—except for incubation with 5-FU and the control (Figure 5a).

The increase in the incubation time after sub-microsecond ECT led to an increase in the frataxin signal (Figure 5b), whereas, in the case of the microsecond protocol, an increase was not observed. At 24 h incubation after nsEP-1, the expression of frataxin increased among all used drugs. However, the highest expression level was observed when 5-FU was combined with iron (III)–EDTA. With the elongated incubation time, the cells treated with 5-FU showed decreased frataxin expression, and the others increased. Concerning nsEP-2 therapy, the iron (III)–EDTA-treated cells revealed the highest frataxin expression, which diminished over time. In comparison, when combining iron (III)–EDTA with 5-FU, the expression was delayed, and the highest expression occurred after 48 h of incubation. Analyzing the µsEP therapy, 5-FU decreased frataxin’s expression and the effect diminished with an elongated incubation time.

## 3. Discussion

The properties of iron-containing compounds highly depend on the pH of the solution [52]. In the case of biological systems, the pH is controlled by various buffers and, therefore, does not change, remaining at 7.4 [53]. Thus, the analysis of the stability of the studied compounds is essential. Two of the analyzed iron supplements differ in their stability constants [54]. Iron (III) citrate is more vulnerable, and, at a non-acidic pH = 7.4, it decomposes into citrate anions and Fe^3+^ cations. The further hydrolysis of metal ions leads to a decrease in free ferric ions and thus shifts the complex’s decomposition further. Conversely, iron (III)–EDTA is highly stable, and its decomposition does not occur. The concentration of a free ligand is sub-micromolar and the vast majority of ferric ions remain complexed. In vitro incubation experiments showed the standalone anticancer potency of the analyzed compounds, applied at high concentrations (>250 μM). At the lower concentrations, each of them exhibited pro-cancerous activity by increasing the viability. The frataxin expression decreased over time, concluding that ferroptosis is not induced by standalone incubation with the analyzed compounds.

At present, the mechanism of action and the interactions of iron complexes in cancer cells are not well clarified. According to complex stability studies, ferric ions and citrate anions act separately on the cell. Although extracellular citrate is proven to inhibit tumor growth and progression, intracellular citrate acts in different ways [55]. Citrate enhances the viability of the cells by delivering the substrate for the Krebs cycle, and ferric ions may affect the cells by delivering the metal cofactor to the iron-dependent enzymes [56]. Iron (III) citrate, when dissociated in the cellular environment, releases ferric ions and citrate anions. Ferric ions can catalyze the formation of reactive oxygen species (ROS) through Fenton reactions, which can induce DNA damage and promote oncogenic transformations. Simultaneously, the citrate anions are metabolized within the Krebs cycle, potentially enhancing the cellular energy production and supporting the increased metabolic demands of proliferating cancer cells. This dual role of iron (III) citrate may not only provide a proliferative advantage but also contribute to the formation of a cellular environment conducive to cancer progression. Furthermore, the availability of ferric ions might affect the activity of iron-dependent enzymes critical for DNA synthesis and repair, further influencing cancer cell survival and proliferation [57]. With the application of iron (III) citrate in combination with 5-FU, the cytotoxic effect of 5-FU decreases. In most combinations, after 24 and 48 h incubation, the interactions between the drugs were found to be antagonistic. The combination of the compounds did not induce an anticancer effect, leading to the conclusion that the possible supplementation of ferric citrate during 5-FU anticancer therapy should be carefully taken into account. Here, the electroporation protocols stimulated a synergistic interaction between the iron compounds and 5-FU. In each case, the compounds did not decrease the progression of frataxin expression over time, meaning that no ferroptosis induction occurred [31].

The Fe(III)–EDTA complex exhibits greater standalone LoVo cell cytotoxicity than iron (III) citrate. Curiously, the combination of the iron (III)–EDTA complex with 5-FU produces worse results than standalone incubation with the iron (III)–EDTA complex. With the increasing concentration of iron (III)–EDTA, the cells’ viability decreases and the effect is exacerbated by the elongation of the incubation time. The viability effect is somewhat dependent on the increase in the 5-FU concentration. Taking into consideration that both of the compounds possess anticancer properties [51], there must be a mechanism in which the 5-FU is neutralized by the iron (III)–EDTA complex [58]. A possible reason for this is the formation of the 5-FU–iron(III)–EDTA complex [58]. The other possibility is that the enhancement of the 5-FU-neutralizing enzymes by the iron (III)–EDTA complex occurs. Thus far, other research has proven the formation of an Fe^3+^–EDTA–uracil complex, but further studies should be conducted in this field to investigate the formation of the Fe^3+^–EDTA–5-FU complex [58].

A promising anticancer effect was induced by nanosecond electric pulses (nsEP), when cells were exposed to iron compounds, 5-FU or a mixture. The mechanism of nsEP includes voltage-mediated shock induction to the cell membrane, without the initial redistribution of the ions across the membrane, which is the case in the microsecond EP protocol [40,59]. High voltages may potentially affect not only the cell membranes but also the added iron-containing compounds. The compounds could undergo reduction on the electrodes to a lower oxidation state (+II) and thus decrease the amount of electricity applied directly to the membrane [60,61]. Iron (III) citrate has been proven to decompose with the release of carbon dioxide when the iron ion is reduced to the second oxidation state [62]. Therefore, the analysis of the permeability of the membranes after electroporation with iron(III) citrate and iron(III)–EDTA (except iron(III)–EDTA in the nsEP-1 protocol) in the nanosecond protocol did not lead to significantly higher permeability than in the control cells. Conversely, in the case of the microsecond EP protocol, the relative permeability was strongly decreased by EP with iron (III) citrate. The differences in the cells’ responses to the citrate and EDTA complex may arise from the differences in their standard reduction potential [54]. Other research provides data indicating that the reduction potential of the EDTA complex (+0.096 V) is significantly lower than that of the free Fe^3+^/Fe^2+^ pair (+0.77 V). For the citrate complex, the standard potential (+0.372 V) remains the highest, meaning that the ferric citrate complex requires a lower voltage to undergo reduction than free Fe^3+^ (as well as its hydrolyzed forms) or the EDTA complex of Fe(III) [63]. This tendency is seen in electro-permeabilization studies, where Fe(III) citrate could prevent the membrane by self-reduction, thus decreasing the effective electric field acting on the plasmalemma of CC cells. Furthermore, the differential activity of iron (III) citrate and iron (III)–EDTA during the electroporation protocols can be attributed to their intrinsic chemical and physical properties, which impact their interactions with cellular components under high-voltage conditions. Iron (III) citrate, possessing a higher standard reduction potential, is more prone to reduction during electroporation. This reduction not only influences its stability but may also reduce the efficacy of the electric field applied during electroporation, thereby modulating the permeability of the cell membranes less effectively than iron (III)–EDTA. In contrast, iron (III)–EDTA, with its lower reduction potential and higher stability, maintains its structural integrity better under similar conditions, thus potentially allowing a more consistent and potent interaction with the cellular machinery. This difference in behavior under electroporation could be crucial in optimizing the application of these compounds in therapeutic settings, suggesting a direction for further investigation into their specific interactions with cellular targets during the electroporation process. According to the viability assay, our study reveals that the combination of 5-FU with any of the iron-containing compounds could be considered cancer-promoting. However, electric pulses significantly reverse this effect. Conversely, the analysis of the frataxin expression leads to the conclusion that there could be a potential application of such combinations. The application of the nsEP-1 protocol induced a progressive decrease in frataxin expression over time after the combination of 5-FU with iron (III) citrate. In the same context, nsEP-2 was revealed to be effective in inhibiting the FXN gene’s expression over time. On the other hand, μsEP with 5-FU with iron–EDTA is effective in decreasing the expression’s progression over time. Moreover, the EP protocol is effective in inducing the instant loss of frataxin expression; however, the procedure does not lead to the inhibition of frataxin’s progression over time. In summary, the combination of 5-FU with iron-containing supplements should not be considered cytotoxic but rather unsuitable for further mitochondria-affecting therapy [64]. In this case, only EP-enhanced drug delivery caused a synergistic drug interaction.

Aside from the combinational therapies, EP with standalone drugs was revealed to be effective in inhibiting frataxin’s expression. In particular, 5-FU mixed with the Fe(III) complexes and supported by electric pulses (nanosecond mainly) was proven effective against colon cancer cells. Moreover, the viability tests proved the high potency of the therapy. A great standalone cytotoxic effect was observed after nsEP2. At lower voltages, the drug only inhibits the temporal progression of frataxin’s expression. At higher ones, the therapy induces a reduction in frataxin expression. In no cases was the standalone iron (III) citrate therapy effective. This could be due to the fact that, with the exception of ferric ions, citrate flows to the cells, enhancing the Krebs cycle rate and supporting cells’ growth with energy [56].

## 4. Materials and Methods

### 4.1. Drug Solution Preparation

Iron (III) citrate (C_6_H_5_FeO_7_) (Sigma-Aldrich, Merck Millipore, Poznan, Poland), EDTA iron (III) sodium salt ([(O_2_CCH_2_)2NCH_2_CH_2_N(CH_2_CO_2_)_2_]FeNa·xH_2_O) (Sigma-Aldrich, Merck Millipore, Poznan, Poland) and 5-fluorouracil (Sigma-Aldrich, Merck Millipore, Poznan, Poland) were dissolved in miliQ water to prepare the stock of the drug. Subsequently, the proper amount of stock was mixed with DMEM or electroporation SKM buffer to achieve the required concentration of the drug. A new drug stock was produced before each experiment.

The chemical analysis of the composition of the iron (III) citrate and iron (III)–EDTA solutions was performed.

Citric acid hydrolysis constants: pK_a1_ = 3.13, pK_a2_ = 4.76, pK_a3_ = 6.4 [65].

All in vitro experiments were performed under pH = 7.4 and room temperature (25 °C), so that the calculations are conducted in standard biochemical conditions. The stability constants (Table 4) were not affected by the pH, but the free Fe^3+^ hydrolyzed, thus decreasing the amount of the complexed iron form in the solution [53]. The total amount of iron could be described by the molar fraction formulas

$$C_{Fe}=\left[Fe^{3+}\right]+\left[FeOH^{2+}\right]+\left[Fe{{\left(OH\right)}_{2}}^{+}\right]+\left[Fe{\left(OH\right)}_{3\;\left(aq\right)}\right]+\left[Fe{{\left(OH\right)}_{4}}^{-}\right]+\left[complex\right]$$

$$C_{ligand}=\left[complex\right]+\left[ligand\right]+\left[Hligand^{+}\right]+\left[H_{2}ligand^{2+}\right]+\left[H_{3}ligand^{3+}\right]+\left[H_{4}ligand^{4+}\right]$$

Given that the ligand-to-metal ratio was consistently 1:1 in the compounds studied, the formulas mentioned are equivalent to

$$C_{Fe}=C_{ligand}$$

$$C_{Fe}=\left[Fe^{3+}\right]+\frac{\left[Fe^{3+}\right]\times \beta_{1}}{\left[H^{+}\right]}+\frac{\left[Fe^{3+}\right]\times \beta_{2}}{{\left[H^{+}\right]}^{2}}+\frac{\left[Fe^{3+}\right]\times \beta_{3}}{{\left[H^{+}\right]}^{3}}+\frac{\left[Fe^{3+}\right]\times \beta_{4}}{{\left[H^{+}\right]}^{4}}+\left[complex\right]$$

Based on this,

$$0=\frac{\beta_{complex}}{k}\times {\left[Fe^{3+}\right]}^{2}+\left(1+\frac{\beta_{1}}{\left[H^{+}\right]}+\frac{\beta_{2}}{{\left[H^{+}\right]}^{2}}+\frac{\beta_{3}}{{\left[H^{+}\right]}^{3}}+\frac{\beta_{4}}{{\left[H^{+}\right]}^{4}}\right)\times \left[Fe^{3+}\right]-C$$

where *k* for the EDTA complex is

$$k=\frac{1+\frac{\left[H^{+}\right]}{K_{a4}}+\frac{{\left[H^{+}\right]}^{2}}{K_{a4}K_{a3}}+\frac{{\left[H^{+}\right]}^{3}}{K_{a4}K_{a3}K_{a2}}+\frac{{\left[H^{+}\right]}^{4}}{K_{a4}K_{a3}K_{a2}K_{a1}}}{1+\frac{\beta_{1}}{\left[H^{+}\right]}+\frac{\beta_{2}}{{\left[H^{+}\right]}^{2}}+\frac{\beta_{3}}{{\left[H^{+}\right]}^{3}}+\frac{\beta_{4}}{{\left[H^{+}\right]}^{4}}}$$

and *k* for the citrate complex is

$$k=\frac{1+\frac{\left[H^{+}\right]}{K_{a4}}+\frac{{\left[H^{+}\right]}^{2}}{K_{a4}K_{a3}}+\frac{{\left[H^{+}\right]}^{3}}{K_{a4}K_{a3}K_{a2}}}{1+\frac{\beta_{1}}{\left[H^{+}\right]}+\frac{\beta_{2}}{{\left[H^{+}\right]}^{2}}+\frac{\beta_{3}}{{\left[H^{+}\right]}^{3}}+\frac{\beta_{4}}{{\left[H^{+}\right]}^{4}}}$$

The results derived from solving the equations were graphed to examine the overall distribution of the iron forms in the test solutions.

### 4.2. Cell Culture

The LoVo cell line (Dukes’ type C, grade IV colon cancer) was obtained from the left supraclavicular metastatic region of a 56-year-old male patient (ATCC^®^, London, UK). The cells were grown in a monolayer cultured in Nutrient Mixture F-12 Ham (Sigma, Merck Millipore, Poznan, Poland) supplemented with 10% fetal bovine serum (FBS, Gibco, Thermo Fisher Scientific, Warsaw, Poland) and 1% BioMyc-1 (Sigma-Aldrich, Merck-Millipore, Poznan, Poland) antibiotics. The cells were grown under standard culture conditions at 37 °C in a humidified atmosphere containing 5% CO_2_. When needed, the cells were rinsed with phosphate-buffered saline (PBS, Bioshop, Mainway, Canada) and removed by trypsinization (0.025% trypsin and 0.02% EDTA; Sigma-Aldrich).

### 4.3. MTT Viability Assay

For further viability experiments, the cells were seeded on 96-well plates (Thermo Fisher, Warsaw, Poland) at a count of 2 × 10^4^ cells per well and incubated overnight in a complete growth medium to allow for cell attachment. Then, the drug-free medium was replaced with medium containing drugs (10, 25, 50, 100, 250, 500, 750, 1000 μM of iron (III) citrate or iron (III)–EDTA and 0.01, 0.5, 1, 10, 50 μM of 5-FU). A combination of iron-containing drugs (250, 500, 750 μM) and 5-FU (0.1, 0.5, 1, 10 μM) was also performed. Afterward, the cells were incubated for 24 and 48 h to assess the viability changes over time. The experiment was repeated a minimum of three times.

To perform the MTT assay, the culture medium was removed from the wells and 100 μL of 0.5 mg/mL MTT (Sigma Aldrich) solution in PBS buffer was added to the 96-well plates; for the 6-well plates, the final volume was 0.2 mL. After 2 h incubation at 37 °C, acidified isopropanol (100 μL, 0.04 M HCl in 99.9% isopropanol) was added to dissolve the formazan crystals. The absorbance of each well was measured at 570 nm using a multiplate reader (GloMax ® Explorer, Promega, GmbH, Walldorf, Germany). The results were expressed as the percentage of viable cells relative to untreated control cells.

### 4.4. Cell Membrane Permeabilization—Flow Cytometry Studies of Yo-Pro-1 Uptake

The efficiency of cell membrane permeability in response to the electric field was analyzed by flow cytometry using a fluorescence-activated cell sorter (Cube-6, SYSMEX EUROPE GmbH, Warsaw, Poland). Cells were detached from the culture flasks with trypsin, centrifuged and suspended in low-conductivity (0.12 S/m) electroporation phosphate SKM buffer (10 mM KH_2_PO_4_/K_2_HPO_4_, 1mM MgCl_2_, 250 mM sucrose, pH 7.4) [70] at a total of 2 × 10^5^ cells per cuvette (two aluminum plate electrodes, 2 mm gap). For each sample, iron (III) citrate or iron (III)–EDTA in an appropriate amount was added before the EP protocol. Afterward, the cells were electroporated using the following protocols: E0—no electroporation; nsEP1—30 kV/cm, 400 pulses, 20 ns, 100 Hz; nsEP2—60 kV/cm, 400 pulses, 20 ns, 100 Hz; ESOPE—1200 V/cm, 8 pulses, 100 µs, 1 Hz. Then, the cells were incubated for 20 min at 37 °C in a humidified atmosphere containing 5% CO_2_. In the next step, cells were washed in PBS, centrifugated and resuspended in 0.5 mL of PBS. Flow cytometry analysis was performed using a Cube 6 flow cytometer (Sysmex, Warsaw, Poland). The fluorescence of Yo-Pro-1 was excited with a 488 nm laser and measured with the FL-3 detector (700/50). For each sample, 10^4^ events were analyzed. Data were collected and analyzed using the CyView software (Sysmex, Warsaw, Poland). Measurements were performed in triplicate.

### 4.5. ECT Experiment

EP was performed with the use of the BTX830 (Harvard, MA, USA) electroporator for microsecond pulses or the PPG-20 generator (FID GmbH Technology, Burbach, Germany) for nanosecond pulses, and with cuvettes with two aluminum plate electrodes separated by a 4 mm gap (VWR). The parameters of the experiments are described in Section 4.4. The European Standard Operating Procedures of Electrochemotherapy (ESOPE) protocol was evaluated as well [46,67]. Before each experiment, the apparatus was calibrated to provide the exact electric field. The experiment was monitored, and the generated electric fields that differed from the set by more than 3 V/cm were eliminated. For electroporation, the following parameters we applied: nsEP1—30 kV/cm, 400 pulses, 20 ns, 100 Hz; nsEP2—60 kV/cm, 400 pulses, 20 ns, 100 Hz; ESOPE—1200 V/cm, 8 pulses, 100 µs, 1 Hz. Further, the electroporation buffer was washed out and the cells were seeded on 96-well plates at a total of 2 × 10^4^ cells per well for further viability experiments. The MTT assay was carried out after 24 and 48 h incubation. All experiments were performed using triplicate technical and biological repetitions.

### 4.6. Confocal Microscopy Immunofluorescence Studies

To assess the frataxin content in the cells after ECT with 5-FU, iron (III) citrate and iron (III)–EDTA, as well as after ECT with 5-FU + iron (III) citrate and 5-FU + iron (III)–EDTA, staining with an anti-frataxin antibody was performed. The therapies were performed and the cells were incubated on cover glasses in Petri dishes for 24 and 48 h incubation times. Two incubation times were chosen to investigate the progressive expression of frataxin. The staining procedure involved formalin fixation. One-hour incubation with FBS was performed. After this, 1% Triton-X was added for 10 min to permeabilize the cells. Afterward, the anti-frataxin antibody (anti-frataxin Alexa Fluor^®^ 488, Abcam, ab156033) was added and the samples were left in the incubator for 1 h incubation. At the end, the samples were washed to remove free antibodies and mounted with a DAKO mounting medium. The samples were observed (λ_Ex_: 546 nm, λ_Em_: 570 nm) in the Olympus FluoView FV1000 confocal laser scanning microscope (Olympus, Tokyo, Japan).

### 4.7. Statistical Analysis

Microscopy photographs were analyzed with ImageJ, and the statistical analysis (T-test) was performed using GraphPad Prism version 7.05 for Windows and the GraphPad Software 8.0 (San Diego, CA, USA, www.graphpad.com). The viability experiments were performed in at least 3 replicates. Data were expressed as the mean ± SD and analyzed by multiple t-tests (in GraphPad Prism 7), with *p* < 0.05 considered statistically significant. Drug interactions and combination indexes were calculated in the CompuSyn 1.0 software (https://www.combosyn.com).

## 5. Conclusions

In summary, the use of iron (III) citrate and iron (III)–EDTA as standalone therapies should be seriously considered in anti-colon cancer treatment. Primarily, the low absorption rates of these drugs reduce their efficacy, while their pro-cancerous effects at low concentrations pose significant risks. These issues could potentially be mitigated by incorporating electrochemotherapy (ECT), which enhances drug uptake and effectiveness. Notably, ECT using iron (III)–EDTA in a nanosecond protocol has shown promise, unlike iron (III) citrate, suggesting a differential interaction with the electrical component of the therapy.

Furthermore, the chemotherapy agent 5-fluorouracil (5-FU) has demonstrated effectiveness when used with nsECT and Fe(III). This combination has been found to decrease frataxin’s expression significantly during a microsecond electroporation protocol. However, it is crucial to note that while this approach affects gene expression, it does not substantially impact the viability of cancer cells, indicating that the further optimization of the protocol and dosage might be necessary.

Future research should focus on optimizing the parameters of electroporation, exploring the synergistic effects of drug combinations, and conducting comprehensive clinical trials to evaluate the safety and efficacy of these approaches in a clinical setting. Specifically, trials should assess different combinations and dosages of iron (III) citrate, iron (III)–EDTA and 5-FU under various electroporation conditions to determine the most effective therapeutic regimen to induce cancer cell death while preserving the healthy tissue’s viability. Understanding the molecular mechanisms of these drugs affecting cancer cells can provide insights into more targeted and effective treatments. This multi-faceted approach will be critical in overcoming the current limitations and enhancing the role of these compounds in colon cancer therapy.

## Figures and Tables

**Table 1:** Iron (III) citrate and iron (III)–EDTA analysis.

	Initial Concentration [µM]	Non-Complexed Iron Concentration [µM]	Complexed Iron Concentration [µM]
Iron (III) citrate	250	249.4859	0.514115313
	500	499.5986	0.401381669
	750	749.7114	0.288648026
Iron (III)–EDTA	250	0.000165	249.9998345
	500	0.000237	499.9997631
	750	0.000287	749.9997129

**Table 2:** Combination index and type of interaction between 5-FU and iron compounds according to MTT after 24 h and 48 h. * Combination index (CI) was calculated using CompuSyn software ver.1.0, where CI = 1, <1 and >1 indicate additive effect (Ad), synergism (S) and antagonism (A), respectively.

Incubation Time [h]	Fe(III) Citrate [µM]	5-FU [µM]	CI *	Interaction Type	Fe (III)–EDTA [µM]	5-FU [µM]	CI *	Interaction Type
24	250	0.1	2.39725	A	250	0.1	4.53018	A
		0.5	2.31710	A		0.5	4.81550	A
		1.0	4.54515	A		1.0	5.10450	A
		10.0	5.57859	A		10.0	7.94892	A
	500	0.1	5.36120	A	500	0.1	8.73229	A
		0.5	4.67136	A		0.5	7.71052	A
		1.0	8.08965	A		1.0	6.82734	A
		10.0	3.18717	A		10.0	7.41533	A
	750	0.1	11.1007	A	750	0.1	6.59179	A
		0.5	6.85439	A		0.5	6.39788	A
		1.0	6.55002	A		1.0	6.20665	A
		10.0	13.1310	A		10.0	6.20246	A
48	250	0.1	2.81920	A	250	0.1	4.55482	A
		0.5	2.47332	A		0.5	4.95817	A
		1.0	2.38075	A		1.0	5.53480	A
		10.0	9.81364	A		10.0	15.5189	A
	500	0.1	4.61703	A	500	0.1	8.31723	A
		0.5	4.92391	A		0.5	7.54574	A
		1.0	5.24156	A		1.0	4.50101	A
		10.0	15.4676	A		10.0	5.76203	A
	750	0.1	9.18624	A	750	0.1	9.40481	A
		0.5	8.98559	A		0.5	8.87111	A
		1.0	7.86185	A		1.0	6.21390	A
		10.0	3.18740	A		10.0	4.86205	A

**Table 3:** Combination index and type of interaction between 5-FU and iron compounds post-EP exposure according to MTT after 24 h and 48 h. * Combination index (CI) was calculated using CompuSyn software ver. 1.0, where CI = 1, <1 and >1 indicate additive effect (Ad), synergism (S) and antagonism (A), respectively.

Fe(III) Citrate 250 [µM] /5-FU 10 [µM]	24 h CI *	48 h CI *	Interaction Type	Fe (III)–EDTA 250 [µM] /5-FU 10 [µM]	24 h CI *	48 h CI *	Interaction Type
EP0	5.57859	9.81364	A	EP0	7.94892	15.5189	A
nsEP1	0.03492	0.47368	S	nsEP1	1.85407	1.79286	A
nsEP2	7.37 × 10^−9^	7.04 × 10^−7^	S	nsEP2	0.02511	0.01177	S
ESOPE	4.84 × 10^−6^	1.80 × 10^−4^	S	ESOPE	0.44229	0.34419	S

**Table 4:** Stability constants (25 °C) of the iron complexes [66,67,68].

$\beta_{Fe3+citrate}=\frac{\left[Fe^{3+}-citrate\right]}{\left[Fe^{3+}\right]\left[citrate\right]}=10^{11.85}$	$Fe^{3+}+citrate\to Fe^{3+}-citrate$
$\beta_{Fe3+-EDTA}=\frac{\left[Fe^{3+}-EDTA\right]}{\left[Fe^{3+}\right]\left[EDTA\right]}=10^{25.10}$	$Fe^{3+}+EDTA\to Fe^{3+}-EDTA$

Fe^3+^ hydrolysis constants: log(β_1_) = −2.19, log(β_2_) = -5.76, log(β_3_) = −14.3 and log(β_4_) = −21.71 [69]. H_4_EDTA hydrolysis constants: pK_a1_ = 2.0, pK_a2_ = 2.7, pK_a3_ = 6.16, pK_a4_ = 10.26 [54].

## Data Availability

Available on request to the corresponding author.
